# Chrysanthemum extract attenuates hepatotoxicity via inhibiting oxidative stress *in vivo* and *in vitro*

**DOI:** 10.29219/fnr.v63.1667

**Published:** 2019-04-15

**Authors:** Zixia Tian, Haiyan Jia, Yuezhen Jin, Minghui Wang, Jiejian Kou, Chunli Wang, Xuli Rong, Xinmei Xie, Guang Han, Xiaobin Pang

**Affiliations:** 1 Pharmaceutical Institute, Henan University, Kaifeng, China; 2 Henan Medical Technician Institute, Kaifeng, China; 3 Kaifeng Key Lab for Application of Local Dendranthema morifolium in Food & Drug, Kaifeng, China

**Keywords:** chrysanthemum extract, liver injury, antioxidative, anti-apoptosis, nuclear factor erythroid-2-related factor 2

## Abstract

**Background:**

‘*Bianliang ziyu*’, a famous chrysanthemum variety commonly planted in Kaifeng, China, is often consumed by local residents. However, the hepatoprotective effects of *Bianliang ziyu* and their underlying mechanisms are not clear.

**Objective:**

In this study, we investigated the hepatoprotective and antioxidative effects of *Bianliang ziyu* extract (BZE) on liver injury and explored its molecular mechanisms.

**Design:**

Sprague-Dawley rats were administered BZE by intragastric administration for 8–9 days, and then alcohol or carbon tetrachloride (CCl_4_) was administered by gavage to induce acute liver injury. The activities of serum alanine aminotransferase, aspartate aminotransferase, superoxide dismutase, and malondialdehyde in the rats were measured, and the liver of each rat was examined for histopathological changes. *In vitro*, HL-7702 cells were pretreated with BZE for 24 h and then exposed to 30 mmol•L^−1^ acetaminophen (APAP) for 12 h. The survival rate of the cells and the alanine aminotransferase and aspartate aminotransferase activities were determined. Then, we investigated the effects of BZE on oxidative stress, apoptosis, and the activation of nuclear factor erythroid-2-related factor 2 (Nrf2) signaling in HL-7702 cells induced by APAP.

**Results:**

The results showed that BZE prevented alcohol-, CCl_4_-, and APAP-induced liver injury and suppressed hepatic oxidative stress *in vitro* and *in vivo*. BZE was also observed to significantly inhibit the reduction of mitochondrial membrane potential and regulate the expression of Bcl-2, Bax and Caspase-3 in APAP-induced HL-7702 cells. In addition, BZE significantly promoted nuclear translocation and the expression of Nrf2 as well as its downstream gene hemeoxygenase-1 (HO-1) *in vitro*. Furthermore, the findings showed that Nrf2 siRNA reversed the effects of BZE on cell survival and apoptosis-related protein expression in APAP-induced HL-7702 cells.

**Conclusions:**

BZE plays an important role in preventing hepatotoxicity by inhibiting oxidative stress and apoptosis through activation of Nrf2 signaling. BZE could be developed as an effective functional food for protecting the liver.

## Popular scientific summary

- In this study, we evaluated the hepatoprotective activity of an extract (BZE) from a traditional ornamental chrysanthemum.
- BZE protected the liver from damage caused by alcohol, CCl4 and acetaminophen via antioxidative effects.
- Furthermore, we found that BZE promoted the Nrf2 signaling pathways and helped regulate apoptosis protein expression, thereby enhancing the ability of cells to resist liver damage.
- Our work is expected to promote BZE as a functional food or medication for liver protection.

## 

The liver is an important organ in the body and plays key roles in regulating many physiological processes (1). It is also a vital organ for metabolizing drugs and toxic chemicals. The hepatocytes that make up most of the structure of the liver are very active during the metabolism of exogenous chemicals, and thus the liver and concomitantly the hepatocytes are a major target of toxic substances (2). Many factors, such as alcohol, carbon tetrachloride (CCl_4_), tert-butyl hydroperoxide, and the excessive use of acetaminophen (APAP), can induce liver damage (3). Liver diseases have become a problem worldwide due to the lack of effective preventive methods and treatment options, which lead to poor prognosis and high mortality (4). Therefore, finding valid treatments is key to addressing the challenges of liver diseases (5).

Much evidence has shown that oxidative stress, which has numerous causes, is an important biological mechanism of liver damage (6, 7). Oxidative reactions in cells mainly occur in mitochondria, which is also the main site of reactive oxygen species (ROS) production (8). When the production of ROS exceeds the scavenging capacity of antioxidant enzymes, it can damage mitochondria and spill into the cytoplasm, resulting in cell damage (9). Mitochondrial damage can trigger the release of caspase-3 into the cytoplasm, resulting in apoptosis (10). Moreover, many studies have shown that apoptosis plays an important role in the etiology of liver disease (11). Members of the Bcl-2 protein family (including anti-apoptotic protein Bcl-2 and apoptotic protein Bax) play key regulatory roles in the process of apoptosis (10).

Nuclear factor erythroid-2-related factor 2 (Nrf2) regulates various antioxidant enzymes, such as heme oxygenase-1 (HO-1), superoxide dismutase (SOD), and glutathione (GSH) (12, 13). Under normal conditions, Nrf2 and its inhibitor (Keap-1) are bound in an inactive state in the cytoplasm, and the dissociation of Nrf2 from Keap-1 and the degradation of Nrf2 are in a dynamic equilibrium (14, 15). When exposed to external stimuli, Nrf2 dissociates from Keap-1 to enter the nucleus and bind to the antioxidant response element (ARE), regulating the expression of its downstream enzyme encoding genes (16). Increasing evidence has shown that the Keap-1/Nrf2 signaling pathway plays a key role in maintaining cellular redox homeostasis, and thus it has become an important target for the prevention and treatment of oxidative stress-related diseases, including liver oxidative stress damage caused by exogenous compounds toxic to the liver (16–18).

In recent years, with the continuous development of traditional Chinese medicine, many natural plants have been shown to have good protective effect on the liver. They are inexpensive, easy to obtain, and have few toxic side effects (19). Natural products extracted from medicinal plants are considered to be effective and safe alternatives to conventional hepatoprotective treatments. Chrysanthemums are used as a traditional Chinese medicine to dispel wind, dissipate heat, clear the liver, and improve eyesight (20). Studies on the chemical composition and pharmacological activities of chrysanthemums have shown that flavonoid compounds are the essential medicinal ingredients of chrysanthemums. These compounds can be used in the treatment of acute or chronic liver injury, soreness, swelling, dizziness, hypertension, coronary heart disease, and other diseases, however, their molecular mechanisms are unclear (21, 22). Flavonoids have strong antioxidant activity, so they can effectively eliminate the free radicals produced in an organism and inhibit the various types of damage caused by excess free radicals in the body (23). Currently, whether the active antioxidants in natural plants can effectively prevent liver injury is an active area of study in the food and medical chemistry fields (24).

Kaifeng’s chrysanthemums from China are known throughout the world. ‘*Bianliang ziyu*’ is a famous variety of this type of chrysanthemum, and they have extremely high ornamental value. This type of chrysanthemum is often consumed by local residents. To further explore its nutritional and medicinal value, researchers from the Medicine Research Institute of Henan University used ethanol extraction to obtain *Bianliang ziyu* extract (BZE), which is rich in flavonoids. The aims of this study were to investigate the hepatoprotective effects of BZE and its antioxidant mechanism based on *in vivo* and *in vitro* models of liver injury. The results of this study may promote *Bianliang ziyu* chrysanthemum as a candidate medicinal plant for the protection of the liver.

## Methods

### Compounds and reagents

CCl_4_ was obtained from Sinopharm Chemical Reagent Co. Ltd. (Shanghai, China); APAP was obtained from Shanghai Aladdin Bio-Chem Technology Co. Ltd. (Shanghai, China); LaiPuRui (LPR) was obtained from Tonghua Renmin Pharmaceutical Co. Ltd. (Jilin, China); RPMI 1640 was purchased from Gibgo Company (Thermo Fisher Scientific, MA, USA); kits for detecting alanine aminotransferase (ALT) and aspartate aminotransferase (AST) activity, ROS, malondialdehyde (MDA), GSH, and SOD were purchased from Nanjing Jiancheng Bioengineering Institute (Nanjing, China); Trolox, DAPI, Triton-100, whole-cell protein extract, penicillin, and streptomycin were purchased from Solarbio Science & Technology Co. (Beijing, China); rhodamine 123 (Rh123), enhanced chemiluminescence kits were purchased from Beyotime Biotechnology (Shanghai, China); Alexa Fluor 488-congugated goat anti-rabbit IgG, Nrf2 rabbit antibody were obtained from Abcam (Cambridge, MA, USA); caspase-3 rabbit antibody, Bax rabbit antibody, and Bcl-2 rabbit antibody were all purchased from Cell Signaling Technology (Danvers, MA, USA); rabbit anti-β-action and anti-rabbit IgG were obtained from Proteintech Group, Inc. (Wuhan, China); Lipofectamine 2000 was bought from Invitrogen (Carlsbad, CA, USA); Nrf2 siRNA was purchased from Generaybiotech Co. Ltd. (Beijing, China). All other reagents used in this study were analytically pure.

### Preparation of BZE

*Bianliang ziyu* chrysanthemums were harvested in Kaifeng, China, and were identified as *Chrysanthemum morifolium* Ramat by Associate Professor Yanli Zhao of Kaifeng Agricultural Science Institute. *Bianliang ziyu* chrysanthemums were dried and ground, and the powder (1.0 g) was refluxed with 40.0 mL of 70% ethanol at 80°C for 2 h. The total flavonoid content in the extract was 30.72% based on ultraviolet spectrophotometric determination. The extract was dissolved in double distilled water and stored at 4°C such that it was always ready for use.

### Animal

Male Sprague-Dawley rats (200–220 g) were purchased from the Center of Experimental Animals of Henan Province (Zhengzhou, China). The rats were kept in a controlled environment (temperature: 25 ± 2°C; humidity: 60 ± 5%; 12 h dark/light cycles). The animals had free access to food and water. All studies were carried out in compliance with the Guidelines for the Care and Use of Laboratory Animals (Ministry of Science and Technology of the People’s Republic of China), and all animal protocols were reviewed and approved by the ethics committee of Henan University.

### Animal models and treatments

The first animal model represented alcoholic liver injury, and the tests were performed as follows. The rats were randomly divided into six experimental groups: control group, alcohol group, alcohol + 110 mg•kg^−1^ BZE group, alcohol + 220 mg•kg^−1^ BZE group, alcohol + 440 mg•kg^−1^ BZE group, and positive control group (LaiPuRui [LPR], 110 mg•kg^−1^) (*n* = 8 per group). Rats in the BZE groups were intragastrically administered BZE for 8 consecutive days. On the 9th and 10th days, alcohol (56% v/v, 1.8 mL•100 g^−1^) was administered intragastrically to the rats after 0.5 h of BZE administration, and an equivalent volume of sodium carboxymethyl cellulose (CMC-Na) solution was provided to the rats in the control group. Serum ALT, AST, SOD, and MDA contents were measured after 12 h using blood samples collected from the tail vein. After blood collection, the animals were sacrificed by cervical dislocation. The liver was removed, and the liver index and histopathology were measured.

The second animal model represented CCl_4_-induced liver injury. The rats were randomly divided into the following six experimental groups: control group, CCl_4_ group, CCl_4_ + 110 mg•kg^−1^ BZE group, CCl_4_ + 220 mg•kg^−1^ BZE group, CCl_4_ + 440 mg•kg^−1^ BZE group, and positive control group (LPR, 110 mg•kg^−1^) (*n* = 8 per group). Rats in the BZE group were intragastrically administered BZE for 9 consecutive days. On the 10th day, 10% CCl_4_ olive oil solution (3.0 mL•kg^−1^) was administered intragastrically to rats after 0.5 h of BZE administration, and an equal volume of CMC-Na was provided to the rats in the control group. Serum ALT, AST, SOD, and MDA contents were measured after 16 h using blood samples. After the blood collection, the animals were sacrificed, the liver was removed, and the liver index and histopathological changes were measured.

The liver index was calculated according to the following formula: liver index (%)=liver weight (g)/rat body weight (g) × 100%.

### Histopathological analysis

The histopathological changes in the liver were observed by hematoxylin and eosin (H&E) staining. In brief, the liver tissues were fixed with 4% polymethanol for 24 h, and the fixed tissues were dehydrated in graded ethanol solutions. Then, the liver tissues were embedded in paraffin and cut into 5 μm sections. The paraffin sections were stained with H&E using a standard protocol. The stained specimens were examined and recorded using a light microscope with a camera.

### Determination of biochemical parameters

ALT and AST are routinely measured to evaluate liver disruption (25). The serum or cellular levels of ALT and AST were measured using commercial kits. The serum or cellular levels of MDA, SOD, and GSH were monitored using commercial kits according to the manufacturer’s instructions. The cellular accumulation of ROS was assayed through DCF-DA oxidation (26). In brief, the prepared cells were preincubated with 10 mmol•L^−1^ DCFH-DA for 30 min at 37°C. ROS formation was quantified by a microplate reader (Thermo, Multiskan Ascent, Franklin, USA) at an excitation wavelength of 488 nm and an emission wavelength of 532 nm. Data are presented as the percentage of ROS production versus the control.

### Cell culture

HL-7702 cells were obtained from the Cell Bank of the Chinese Academy of Sciences (Shanghai, China). The highly differentiated HL-7702 cell line was cultured in RPMI 1640 supplemented with 10% FBS, 100 U•mL^−1^ penicillin, and 100 U•mL^−1^ streptomycin at 37°C in 5% CO_2_ in a humidified incubator.

### Cell viability assay

Cell viability was measured using the (3-[4,5-dimethylthiazol-2-yl]-2,5 diphenyl tetrazolium) MTT method. Briefly, the HL-7702 cells were seeded in 96-well plates at a density of 5 × 10^4^ cells per well for 24 h and pretreated with various concentrations of BZE (50, 100, and 200 μg•mL^−1^) and the positive control Trolox (50 μmol•L^−1^) for 24 h before exposure to 30 mmol•L^−1^ APAP for 12 h. After incubation of the cells, viable cells were stained with 0.5 mg/mL MTT. After 4 h, the supernatant was discarded, and the formazan was dissolved in dimethyl sulfoxide. The absorbance of each well was measured at 570 nm using a microplate reader (Thermo, Multiskan Ascent, USA). The percent cell viability was calculated by comparing absorbance values with those of the control cells.

### Measurement of mitochondrial membrane potential

Rh123 staining was conducted to detect the mitochondrial membrane potential. Rh123 is a fluorescent dye that can be absorbed by mitochondria, and the absorbing ability of mitochondria is dependent on its transmembrane potential. HL-7702 cells were seeded in 6-well plates and washed with PBS. Rh123 (5 mg•mL^−1^) stored at −20°C and dissolved in PBS was added to the cell cultures, which were diluted with FBS-free RPMI 1640. Then, the cells were incubated at 37°C for 30 min. The plate was gently washed three times with PBS. The cells were harvested and then resuspended in 500 mL of PBS for the detection of the mitochondrial membrane potentials, which were determined by flow cytometry and expressed as the mean fluorescence intensity of 10,000 cells. The peak area size of each group represents the fluorescence intensity (27).

### Determination of Nrf2 immunofluorescence

HL-7702 cells were seeded in 6-well culture plates at a density of 5 × 10^5^ cells per well. Cells were pretreated with 200 μg•mL^−1^ BZE for 24 h and then fixed in 4% paraformaldehyde solution in PBS for 10 min at room temperature. After washing with PBS, the cells were permeabilized with 0.5% Triton X-100 in PBS for 20 min. After incubation with blocking buffer (4% BSA in PBS) for 1 h, the cells were incubated with a rabbit Nrf2 antibody (1:100) at 4°C overnight and then incubated with an Alexa Fluor 488-conjugated goat anti-rabbit IgG secondary antibody at room temperature for 2 h. The cell nuclei were stained with DAPI (5 μg•mL^−1^ for 10 min). The samples were then examined using a fluorescence microscope (NIKON Eclipse TS100, Nikon, Tokyo, Japan).

### Nrf2 siRNA transfection

Lipofectamine 2000 was used for transfection according to the manufacturer’s instructions. HL-7702 cells were inoculated in a 6-well plate at a concentration of 3 × 10^5^ cells/mL. The cells were transfected with Lipofectamine 2000 with transfection reagent using a total of 100 pmol of RNA per well of a 6-well plate. After transfection for 48 h, the Nrf2 siRNA levels of the cells were measured.

### Western blot analysis

Protein concentrations were measured using a protein assay kit according to the manufacturer’s instructions. Extracted proteins were subjected to sodium dodecyl sulfate polyacrylamide gel electrophoresis (SDS-PAGE) and transferred to polyvinylidene difluoride (PVDF) membranes. Then, each PVDF membrane was blocked with 1 × TBST containing 5% nonfat milk for 2 h at room temperature, and the membranes were incubated with primary antibodies against rat Nrf2, HO-1, Bcl-2, Bax, caspese-3, and β-actin overnight at 4°C. Subsequently, the membranes were washed with TBST and incubated with secondary antibody for 2 h. Blots were developed using an enhanced chemiluminescence (ECL)-plus kit. ImageJ2X was used for gray value analysis.

### Statistical analysis

Data are expressed as the mean ± SD. Differences between groups were examined through one-way analysis of variance (ANOVA), with post hoc analysis conducted using Student’s t-test. The statistics were analyzed by GraphPad Prism 5 software (GraphPad Software, Inc., San Diego, CA, USA). Values of P < 0.05 were considered to be significant.

## Results

### The protective effects of BZE against liver injury in vivo and in vitro

**Fig. 1:**
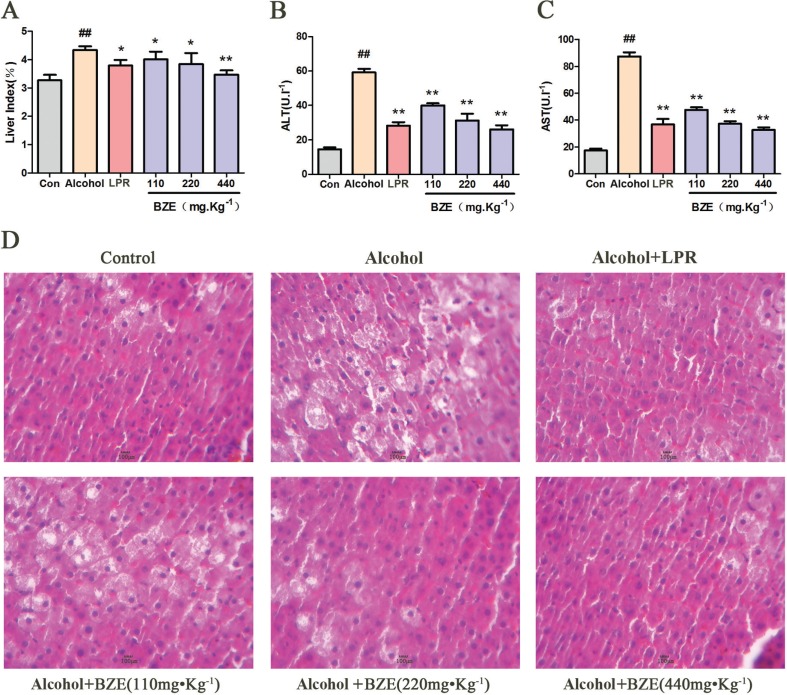
BZE attenuated alcohol-induced hepatic injury in rats. Rats in BZE group were intragastrically administered BZE for 8 consecutive days. On the 9th and 10th days, alcohol (56%v/v, 1.8 ml•100g^−1^) was administered intragastrically to rats after 0.5 h of administration BZE. (A) Effects of BZE on liver index. (B) Effects of BZE on serum ALT activities. (C) Effects of BZE on serum AST activities. (D) Effects of BZE on histopathological changes in rat liver (HE-staining). Typical images are chosen from each experimental group. Data are expressed as means ± SD from three independent experiments. Significant difference was defined as ^##^ *P* < 0.01 vs. control; *P < 0.05, **P < 0.01 vs. alcohol.

We first determined the protective effect of BZE against acute alcoholic liver injury in rats. As shown in Fig. 1A–D, alcohol caused clear increases in the liver index and the levels of serum ALT and AST in the rats. Hepatocellular degeneration effects, including hepatocyte swelling, cytoplasmic porosity, and inflammatory cell infiltration, were observed in the alcohol group. Compared with the alcohol group, BZE significantly reduced the liver index and decreased the levels of serum ALT and AST in the rats. Histopathological changes in the livers of the rats of the BZE groups were also improved significantly with increasing concentration of BZE.

**Fig. 2:**
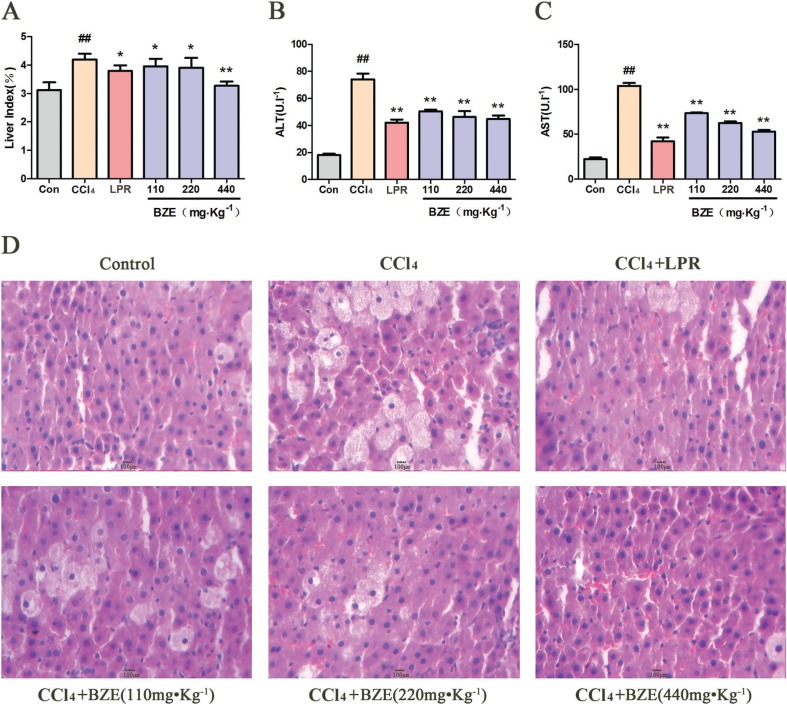
BZE attenuated CCl_4_-induced hepatic injury in rats. Rats in BZE group were intragastrically administered BZE for 9 consecutive days. On the 10th day, 10% CCl_4_ olive oil solution(3.0 ml•kg^−1^) was administered intragastrically to rats after 0.5 h of administration of BZE. (A) Effects of BZE on liver index. (B) Effects of BZE on serum ALT activities. (C) Effects of BZE on serum AST activities. (D) Effects of BZE on histopathological changes in rat liver (HE-staining). Typical images are chosen from each experimental group. Data are expressed as means ± SD from three independent experiments. Significant difference was defined as ^##^ *P* < 0.01 vs. control; *P < 0.05, **P < 0.01 vs. CCl_4_.

Similar results were observed in rats with liver injury induced by CCl_4_ (Fig. 2A–D). Compared with the control group, the liver index and serum levels of ALT and AST were all significantly higher in the CCl_4_ group. The major histopathological change in the model group was more obvious. BZE significantly reduced the liver index, serum ALT, and AST in rats and reduced the hepatocellular degeneration and inflammatory cell infiltration induced by CCl_4_.

**Fig. 3:**
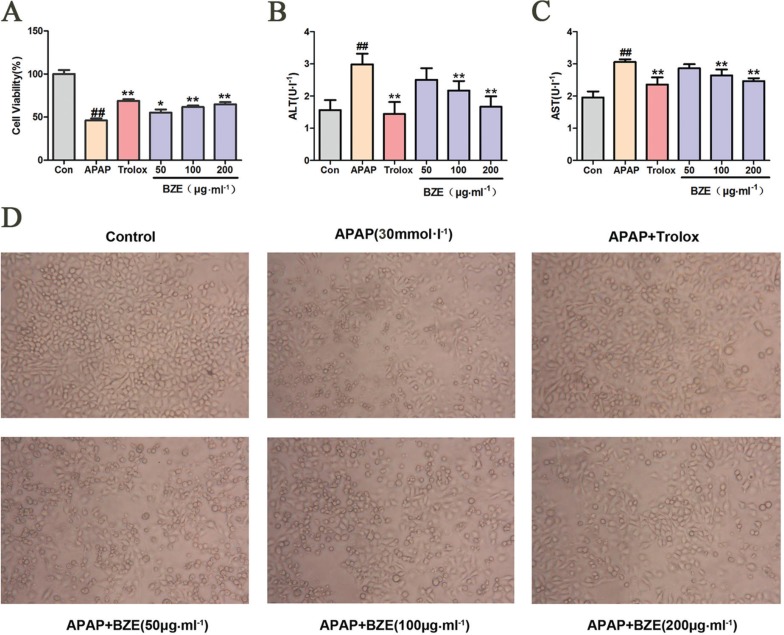
BZE attenuated the toxicity of APAP-induced HL-7702 cells. HL-7702 cells were pretreated with various concentrations of BZE (50, 100, and 200 μg•mL^−1^) and Trolox (50 μmol•L^−1^ ) for 24 h, followed by 12 h of 30 mmol•L^−1^ APAP incubation. (A) BZE increased the cell viability in APAP-treated HL-7702 cells. B-C Effects of BZE on ALT(B) and AST(C) in HL-7702 cells induced by APAP. (D) Effects of BZE pretreatment and APAP exposure on the morphological changes of HL-7702 cells (200×). Typical images are chosen from each experimental group. Data are expressed as means ± SD from three independent experiments. Significant difference was defined as ^##^ *P* < 0.01 vs. control; *P < 0.05, **P < 0.01 vs. APAP.

*In vitro*, Hl-7702 cells were exposed to APAP (30 mmol•L^−1^) to induce hepatocellular injury. As shown in Fig. 3A, APAP decreased cell viability in HL-7702 cells compared to the control, but treatment with BZE increased cell viability in APAP-treated HL-7702 cells in a concentration-dependent manner. In addition, the levels of AST and ALT in HL-7702 cells were significantly increased after exposure to APAP for 12 h. However, pretreatment with BZE significantly attenuated the increase in the ALT and AST levels compared with that seen in the APAP-treated group (Fig. 3B and C). Clear morphological changes were observed in cell cultures exposed to APAP. Cells displayed prominent shrinkage, and the occurrence of nuclear condensation decreased. The number of adherent cells decreased, and the number of detached cells and cell debris significantly increased. However, BZE pretreatment reduced HL-7702 cell morphological deterioration in a concentration-dependent manner (Fig. 3D).

### BZE inhibited oxidative stress in the liver injury model

**Fig. 4:**
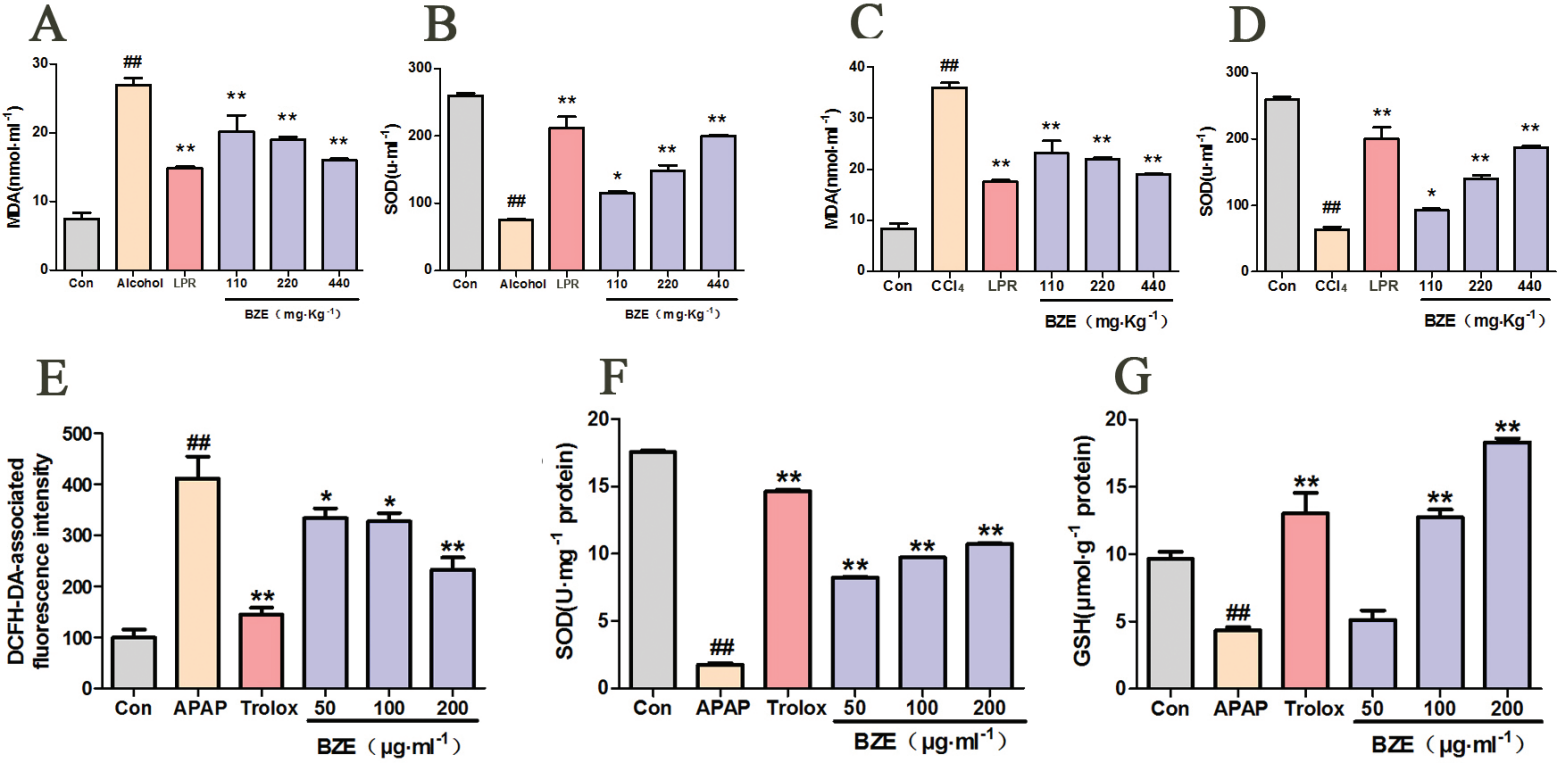
Effects of BZE on the oxidative stress in the liver injury model *in vivo* and *in vitro*. (A–B) Rats were intragastrically administered BZE for 8 consecutive days. On the 9th and 10th days, alcohol (56%v/v, 1.8 ml•100g^−1^) was administered intragastrically to rats after 0.5 h of administration of BZE; serum MDA content (A) and SOD activity (B) were measured. Data are expressed as means ± SD from three independent experiments. ^##^ *P* < 0.01 vs. control; *P < 0.05, **P < 0.01 vs. alcohol group. (C–D) Rats were intragastrically administered BZE for 9 consecutive days. On the 10th day, 10% CCl_4_ olive oil solution (3.0ml•kg^−1^) was administered intragastrically to rats after 0.5 h of administration of BZE; serum MDA content (C) and SOD activity (D) were measured. Data are expressed as means ± SD from three independent experiments. ^##^ *P* < 0.01 vs. control; *P < 0.05, **P < 0.01 vs. CCl_4_ group. (E–G) HL-7702 cells were pretreated with BZE (50, 100, and 200 μg•mL^−1^) and Trolox (50 μmol•L^−1^ ) for 24 h, followed by 12 h of 30 mmol•L^−1^ APAP incubation; cellular ROS production (E), SOD activity (F), and MDA content (G) were measured. Data are expressed as means ± SD from three independent experiments. ^##^ *P* < 0.01 vs. control; *P < 0.05, **P < 0.01 vs. APAP.

Numerous studies have suggested that the levels of ROS, MDA, SOD, and GSH are key indicators of oxidative stress (28, 29). Therefore, we measured serum MDA and SOD levels in rats with liver injury caused by alcohol. As shown in Fig. 4A and B, in the alcohol group, the serum MDA content was higher, and the SOD activity was significantly lower compared with the control group. In contrast, BZE decreased the serum MDA content and increased the SOD activity compared with the alcohol group (Fig. 4A and B).

Next, we performed the same test in rats with liver injury induced by CCl_4_. Similarly, CCl_4_ increased the serum MDA content and decreased the SOD activity in rats. In contrast, the serum MDA content was decreased, and the SOD activity was significantly increased in the BZE-treated group (Fig. 4C and D).

To further investigate the protective effects of BZE against oxidative stress, intracellular ROS levels were assessed in HL-7702 cells exposed to APAP. APAP exposure markedly increased ROS levels by 4.12-fold compared with those in the controls. In contrast, BZE pretreatment decreased this elevation in a dose-dependent manner (Fig. 4E). Since SOD and GSH are important endogenous antioxidants, SOD and GSH deficiencies increase ROS-induced toxicity (30). Therefore, SOD activity and GSH content were measured in BZE-pretreated cells following APAP treatment. APAP exposure significantly reduced the SOD activity and GSH levels in these cells, while BZE pretreatment attenuated these effects in a dose-dependent manner (Fig. 4F and G).

### Effects of BZE on the APAP-induced mitochondrial apoptosis pathway

**Fig. 5:**
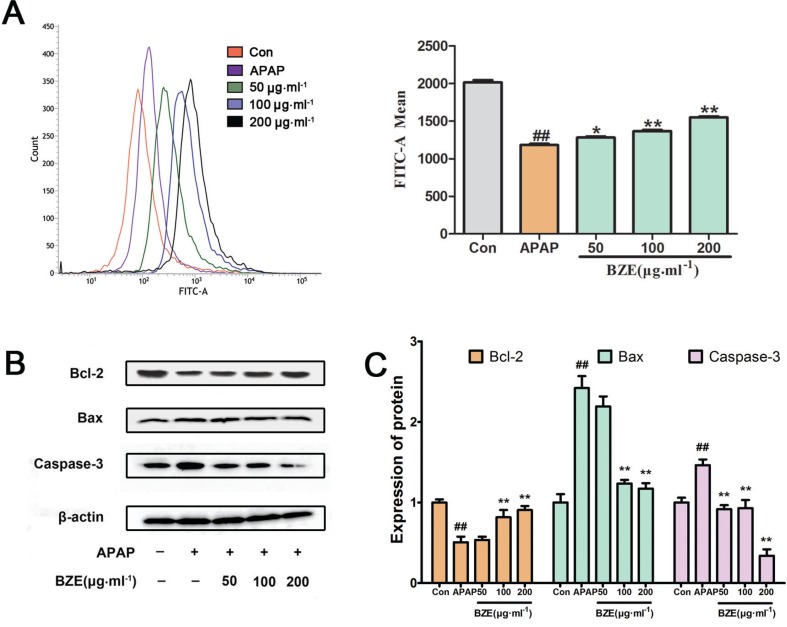
Effects of BZE on APAP-induced mitochondrial membrane permeability and mitochondrial apoptosis pathway. (A) HL-7702 cells were pretreated with BZE (50, 100, and 200 μg•mL^−1^) for 24 h, followed by 12 h of 30 mmol^−1^ APAP incubation, and then stained with rhodamine 123 for 30 min for determination of mitochondrial membrane permeability. Mitochondrial membrane permeability was measured by using flow cytometry and expressed as the mean fluorescence intensity of 10,000 cells. The peak area size of each group represents the fluorescence intensity. (B) Western blot analysis was performed using antibodies against Bcl-2, Bax, and Caspase-3. (C) Quantification of Western blot data. Protein expression was normalized to that of β-actin, and relative densities have been normalized against the control group. Data are expressed as means ± SD from three independent experiments. ^##^ *P* < 0.01 compared to control; *P < 0.05, **P < 0.01 compared to APAP.

The mitochondrial apoptosis pathway triggered by oxygen radicals plays an important role in hepatic injury caused by various factors (31, 32). Accordingly, we examined the mitochondrial membrane potential in APAP-induced HL-7702 cells using flow cytometry after staining the cells with Rh123. As shown in Fig. 5A, exposure to APAP significantly reduced the fluorescence intensity, whereas BZE pretreatment increased the fluorescence intensity compared with the APAP-treated group. These results indicate that BZE inhibits the APAP-induced reduction of the mitochondrial membrane potential. Next, we measured the expression of Bcl-2, Bax, and caspase-3 by Western blotting. Our results showed that the Bcl-2 levels in HL-7702 cells were significantly reduced by APAP treatment compared to those in control cells without APAP, but BZE pretreatment increased the expression of Bcl-2 in a dose-dependent manner (Fig. 5B and C). On the other hand, the expression levels of Bax and caspase-3 were significantly increased in the APAP-treated group compared to those not exposed to APAP. However, the levels of Bax and caspase-3 decreased in the BZE + APAP groups in a dose-dependent manner (Fig. 5B and C).

### BZE promoted the Nrf2 signaling pathway in APAP-induced HL-7702 cells

**Fig. 6:**
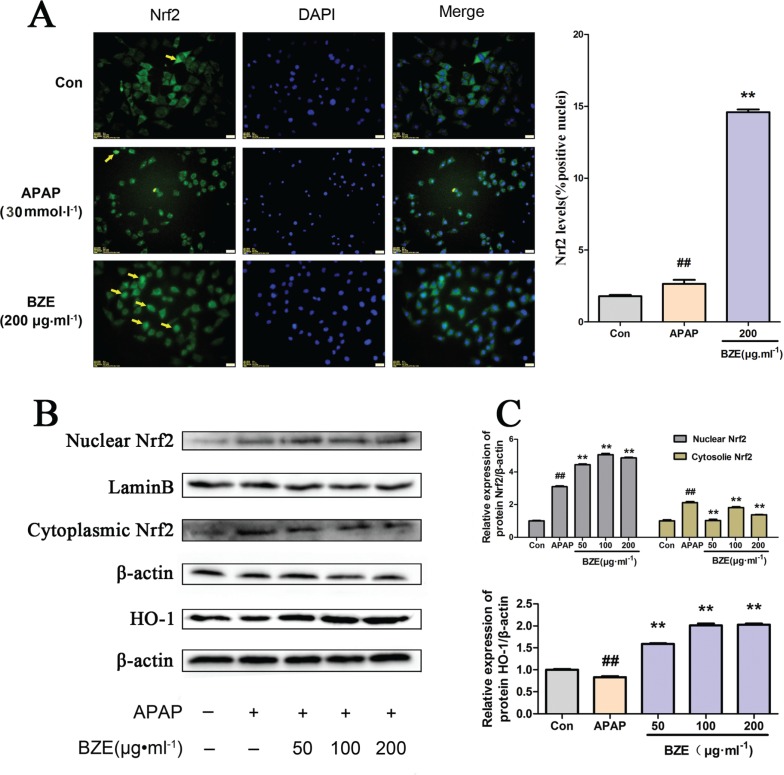
Effects of BZE on Nrf2 pathway activation in APAP-induced HL-7702 cells. (A) BZE promoted translocation of Nrf2 from cytoplasm to nucleus. HL-7702 cells were incubated with BZE (200 μg•mL^−1^) for 24 h, and then Nrf2 localization was observed under an inverted fluorescence microscope. The cells indicated by yellow arrows show Nrf2 translocation to the nucleus, and the bar chart on the right shows the percentage of cells that translocation of Nrf2. (B) Representative Western blotting bands. (C) Relative density analysis of the Nrf2 protein bands in cytosol and nucleus and HO-1 protein. Protein expression was normalized to that of β-actin or Lamin B, and relative densities have been normalized against the control group. Data are expressed as means ± S.D. from three independent experiments.^##^ *P* < 0.01 compared to control; **P < 0.01 compared to APAP.

Nrf2 plays a key role in the ARE-mediated induction of phase II detoxification and antioxidant enzymes (33–35). To investigate the effects of BZE on the Nrf2 pathway, we measured the nuclear translocation of Nrf2 in HL-7702 cells treated with APAP, as well as the expression of Nrf2 and HO-1, the downstream gene of Nrf2, in the same cells. Nrf2 translocation was assessed by fluorescence microscopy. In Fig. 6A, Alexa Flour 488-azide staining (green) indicates the location of Nrf2 antibodies, DAPI staining indicates the location of the nuclei (blue), and the merged image shows the nuclear translocation of the Nrf2 protein. As expected, BZE (200 μg•mL^−1^) promoted translocation of Nrf2 into the nucleus (Fig. 6A). Furthermore, the Western blot results corroborated that the nuclear abundance of Nrf2 was significantly increased by BZE, but the cytoplasmic abundance of Nrf2 was decreased (Fig. 6B and C). In addition, HO-1 protein expression was markedly elevated by BZE (Fig. 6B and C). These results indicate that BZE promotes the activation of the Nrf2 signaling pathway in APAP-injured hepatocytes.

### Nrf2 was involved in the anti-apoptosis effect of BZE as part of its hepatoprotective mechanism

**Fig. 7:**
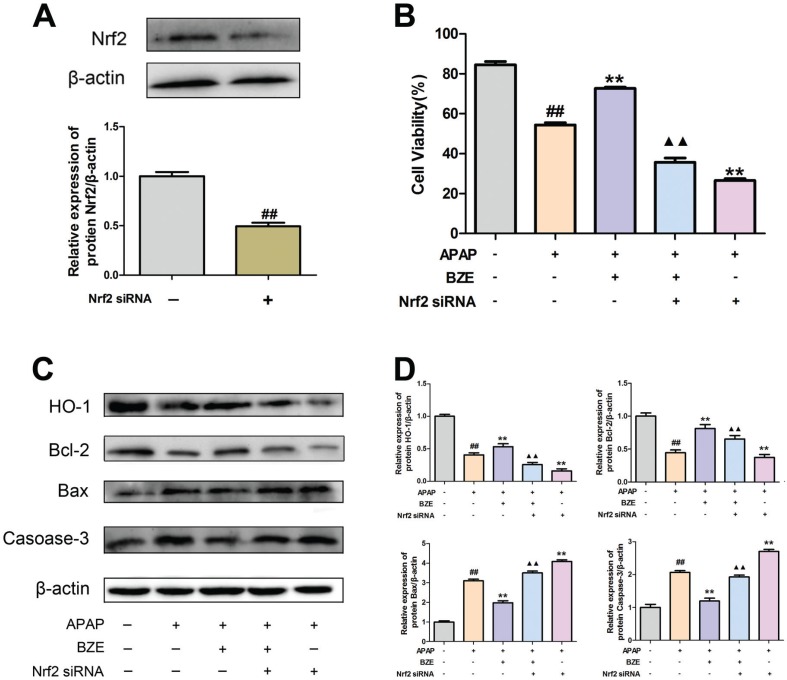
Nrf2 signal activation was involved in anti-apoptosis action induced by BZE. Cells were transfected with Nrf2 siRNA and treated with BZE for 24 h followed by APAP 24 h prior to cell viability measure or Western blot analysis. (A) Cells were transfected with Nrf2-specific siRNA or control siRNA. Downregulation of Nrf2 protein expression by Nrf2 siRNA was confirmed by Western blot analysis. (B) Nrf2 siRNA attenuated the enhancement of cell viability provided by BZE. (C) Representative Western blotting bands. (D) Quantification of Western blot data. Data are expressed as means ± SD from three independent experiments. ^##^ *P* < 0.01 compared to control; **P < 0.01 compared to APAP; ▲▲*P* < 0.01 compared to APAP + BZE.

To examine the association between the Nrf2 signaling pathway and the hepatoprotective effects of BZE, we transfected HL-7702 cells with Nrf2 siRNA and measured the cell survival rate and the expression of apoptosis-related proteins after BZE pretreatment and APAP exposure. Reduced Nrf2 expression was found after the cells were transfected with Nrf2 siRNA (Fig. 7A). In addition, the decreased survival rate after transfection with Nrf2 siRNA suggested that Nrf2 siRNA inhibited the ability of BZE to protect against APAP-induced cytotoxicity in HL-7702 cells (Fig. 7B). In addition, the expression of HO-1, the downstream antioxidant protein, was also decreased. Moreover, Nrf2 siRNA also reversed the impact of BZE on apoptosis-related proteins Bcl-2, Bax, and caspase-3 (Fig. 7C and D). These data suggest that in addition to antioxidative stress, activation of the Nrf2 pathway mediated by BZE also has a crucial anti-apoptotic effect, which are both related to the hepatoprotective effects of BZE.

## Discussion

Chrysanthemums are one of the top 10 most famous flowers in China, and they are well known throughout the world. There are many kinds of chrysanthemums, including medicinal chrysanthemums, edible chrysanthemums, and ornamental chrysanthemums. Medicinal or edible chrysanthemums are often used as dietary supplements because they contain many active ingredients and have a variety of health benefits. An increasing number of studies have shown that chrysanthemums have protective effects toward different types of liver injury, such as chemical liver injury, alcoholic liver toxicity, and drug-induced liver injury (36, 37). However, information on the edibility and liver-protective effects of ornamental chrysanthemums is limited. In this study, BZE, which is an ethanol extract from the *Bianliang ziyu* ornamental chrysanthemum from Kaifeng, China, was investigated for its potential protective effect against alcoholic-, CCl_4_-, and APAP-induced hepatotoxicity. The results showed that BZE exhibited significant protective effects on hepatocytes both *in vivo* and *in vitro*. Moreover, the effects of Nrf2 in mediating antioxidative stress and anti-apoptosis are essential.

The liver is the main organ of metabolism that can store glycogen, detoxify the body, and synthesize plasma proteins. However, the liver is also the main target of toxic substances during oxidative stress (38, 39). The reactive oxygen free radicals generated by the metabolism of ethanol/acetaldehyde cause hepatic tissue injury; CCl_4_ is metabolized by cytochrome P4502E1 (CYP2E1), which generates ROS in the hepatic endoplasmic reticulum and causes damage via lipid peroxidation; APAP is converted in the liver to NAPQI (an active toxic metabolite), which is normally detoxified by GSH. However, the formation of excess NAPQI from APAP can lead to depletion of GSH and oxidative stress injury. Hence, antioxidant effects are key to preventing liver damage from various causes.

The main components of chrysanthemums include volatile oils, flavonoids, amino acids, and trace elements. Flavonoids are known to have antioxidant activities. The total flavonoid content of BZE, the chrysanthemum extract evaluated in this study, reached 30.72%, which is higher than that of traditional medicinal chrysanthemums. In this study, BZE inhibited lipid peroxidation levels and increased the activity of the antioxidant enzyme SOD in rats with liver injury models induced by alcohol and CCl_4_, respectively. In APAP-induced hepatic injury cells, BZE decreased ROS production and enhanced the activities of the antioxidant enzymes GSH and SOD. These results suggest that the antioxidant capacity of BZE protects the liver from hepatotoxicity caused by various factors. The effects of BZE should be attributed to its high flavonoid content.

Oxygen free radicals mainly cause damage by destroying the structure and function of the cell membrane, cause lipid peroxidation; damage to DNA, the mitochondria, and endoplasmic reticulum; and, finally, cause cell death (30). To explore the potential mechanism of BZE’s antioxidant effect on liver injury prevention, we investigated the mitochondrial membrane potential in hepatocytes exposed to APAP. APAP is a widely used antipyretic and analgesic drug. In developed countries, acute liver injury caused by excessive APAP has been reported as the main cause of drug-induced liver injury (40). The underlying mechanism of the hepatotoxicity of APAP is thought to be the conversion of APAP to NAPQI, which can immediately bind to GSH to form a nontoxic metabolite called cysteine. Once the cellular pool of GSH is exhausted, NAPQI covalently binds to cellular proteins, leading to mitochondrial dysfunction and oxidative stress. In this study, we found that APAP induced the reduction of the mitochondrial membrane potential in HL-7702 cells, while BZE increased the mitochondrial membrane potential, suggesting that the liver-protective effect of BZE is related to mitochondrial functions.

Apoptosis is a highly regulated process of programmed cell death. It is regulated by two basic pathways: the death receptor-mediated (external) pathway and the mitochondrial (internal) pathway (41). In the exogenous pathway, the interactions between the ligand and the death receptor (DR) begin with the plasma membrane and then activate caspase-8, which can directly activate the downstream effects of caspase-3 (42). The internal pathways of apoptosis stimulate the permeability of the outer mitochondrial membrane, releasing the dissolved protein into the cytoplasm from the interior of the mitochondria, and activating cysteine aspartic protease to destroy the cell (43). The Bcl-2 family of proteins are regulators of the internal pathways of apoptosis because they control the permeability of the mitochondrial outer membrane in cells (43). The Bcl-2 family of proteins can be divided into three useful subgroups. The first subgroup constitutes the pro-apoptotic proteins (Bax and BAK), which induce mitochondrial outer membrane permeability. The second subgroup is the anti-apoptotic protein (Bcl-2), which can inhibit the membrane permeability of mitochondria. An increase in Bcl-2 expression can alter the internal flow of calcium ions through the mitochondrial membrane, maintain the integrity of the mitochondrial membrane, and regulate the oxidative stress response, thus improving cell apoptosis (44). The third subgroup is the BH3-only protein, which can bind to the apoptotic proteins Bax and BAK and induce a cascade reaction (45). In the present study, we found that APAP significantly increased the expression of the apoptotic proteins Bax and caspase-3 in HL-7702 cells, while the anti-apoptotic protein, Bcl-2, was consumed. BZE pretreatment increased the expression of Bcl-2 and reduced the rise in the Bax and caspase-3 levels. Therefore, BZE may inhibit cell apoptosis after APAP injury via upregulating Bcl-2 and downregulating Bax and caspase-3 expression.

The key role of Nrf2 in preventing APAP-induced hepatotoxicity has been known for decades (46, 47). Nrf2 is an alkaline region-leucine zipper transcription factor that plays a key role in regulating cellular defense against oxidative stress (48). Under normal conditions, Nrf2 is retained in the cytoplasm by binding to the negative regulator Keap1 (49). The production of ROS usually triggers oxidative stress in cells, resulting in the dissociation of Keap1 from Nrf2, followed by Nrf2 translocation into the nucleus, which initiates the expression of antioxidant genes and enzymes by binding to the antioxidant response element (ARE) of the target gene promoter region (50–52). The antioxidant genes activated by Nrf2 include heme oxygenase (HO-1), glutathione (GSH), and superoxide dismutase (SOD), which help protect cells from oxidative stress (53, 54). Our present study showed that APAP exposure resulted in Nrf2 nuclear translocation and upregulation of Nrf2 expression at the protein level, and BZE pretreatment intensified Nrf2 nuclear translocation and protein expression in the nucleus. In the model group, the expression of HO-1 decreased at 12h after APAP injury, which may be due to the fact that APAP was metabolized and consumed a large amount of GSH in the early stage, resulting in a large amount of oxygen free radicals,and HO-1 was rapidly consumed. The change in ROS expression is consistent. The same result is found in other studies (55, 56). In addition, significant upregulation of HO-1 expression occurred in BZE + APAP-treated cells. These data suggest that BZE promotes the activation of Nrf2/HO-1 signaling pathways and accelerates the dissociation of Nrf2 from Keap1, resulting in the expression of cytoprotective protein and upregulation of the induction of cellular antioxidant defense systems, which play important roles in resisting APAP-induced oxidative stress in hepatocytes.

To clarify the effect of Nrf2 on the anti-apoptosis effects of BZE, we compared the cytotoxicity and apoptosis-related protein expression in wild-type and Nrf2 siRNA-transfected cells after APAP treatment. After transfection of Nrf2 siRNA, the APAP-mediated cytotoxicity was worse. The decrease in Bcl-2 expression and the increases in Bax and caspase-3 in the APAP-induced HL-7702 cells were more significant. In addition, Nrf2 siRNA partly reversed the regulatory effects of BZE (2 00 μM) in the above apoptosis-related protein expression. Taken together, these results suggest that activation of the Nrf2 pathway is involved in the anti-apoptosis effects of BZE in HL-7702 cells treated with APAP.

## Conclusion

**Fig. 8:**
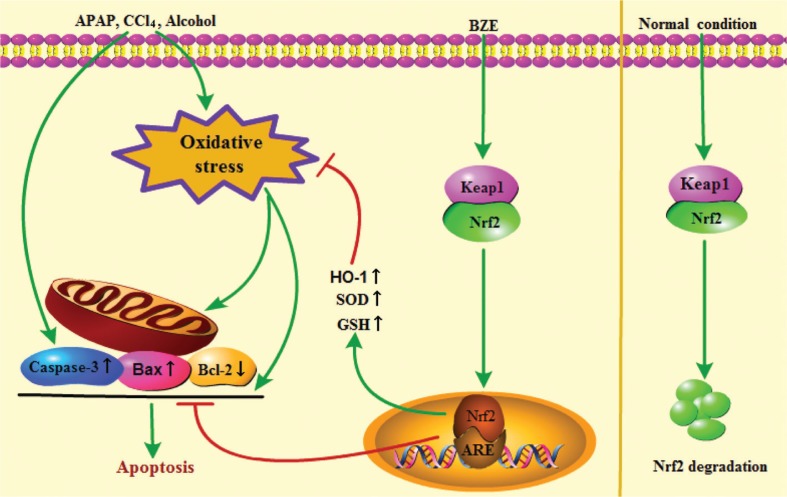
Model description of the protection of chrysanthemum extract BZE against liver damage. BZE promotes the activation of Nrf2 signaling pathway, upregulates the expression of antioxidant enzymes, or participates in the regulation of apoptosis genes expression, thereby enhancing the ability of cells to resist liver damage.

Our work explored a chrysanthemum extract. BZE exerts antioxidant and anti-apoptosis effects against hepatocyte toxicity induced by alcohol, CCl_4,_ or APAP, and the protective effects of BZE are related to the activation of the Nrf2 signaling pathway (Fig. 8). The results of this study can promote the use of the *Bianliang ziyu* chrysanthemum as a medicinal and edible plant for the protection of the liver.
